# α-Fe_2_O_3_ Nanoparticles/Vermiculite Clay Material: Structural, Optical and Photocatalytic Properties

**DOI:** 10.3390/ma12111880

**Published:** 2019-06-11

**Authors:** Marta Valášková, Jonáš Tokarský, Jiří Pavlovský, Tomáš Prostějovský, Kamila Kočí

**Affiliations:** 1 Institute of Environmental Technology, VŠB-Technical University of Ostrava, 17. listopadu 2172/15, 708 00 Ostrava—Poruba, Czech Republic; jonas.tokarsky@vsb.cz (J.T.); tomas.prostejovsky@vsb.cz (T.P.); kamila.koci@vsb.cz (K.K.); 2 Nanotechnology Centre, VŠB-Technical University of Ostrava, 17. listopadu 2172/15, 708 00 Ostrava—Poruba, Czech Republic; 3 Department of Chemistry, Faculty of Materials Science and Technology, VŠB-Technical University of Ostrava, 17. listopadu 2172/15, 708 00 Ostrava—Poruba, Czech Republic; jiri.pavlovsky@vsb.cz

**Keywords:** α-Fe_2_O_3_ photocatalysts, clay materials, photocatalytic activity, molecular simulation

## Abstract

Photocatalysis is increasingly becoming a center of interest due to its wide use in environmental remediation. Hematite (α-Fe_2_O_3_) is one promising candidate for photocatalytic applications. Clay materials as vermiculite (Ver) can be used as a carrier to accommodate and stabilize photocatalysts. Two different temperatures (500 °C and 700 °C) were used for preparation of α-Fe_2_O_3_ nanoparticles/vermiculite clay materials. The experimental methods used for determination of structural, optical and photocatalytic properties were X-ray fluorescence (ED-XRF), X-ray diffraction (XRD), scanning electron microscopy (SEM) with energy dispersive X-ray spectrometry (EDS), N_2_ adsorption method (BET), diffuse reflectance UV-Vis spectroscopy (DRS), photoluminescence spectroscopy (PL) and photocatalytic reduction of CO_2_, respectively. The data from XRD were confronted with molecular modeling of the material arrangement in the interlayer space of vermiculite structure and the possibility of anchoring the α-Fe_2_O_3_ nanoparticles to the surface and edge of vermiculite. Correlations between structural, textural, optical and electrical properties and photocatalytic activity have been studied in detail. The α-Fe_2_O_3_ and α-Fe_2_O_3_/Ver materials with higher specific surface areas, a smaller crystallite size and structural defects (oxygen vacancies) that a play crucial role in photocatalytic activity, were prepared at a lower calcination temperature of 500 °C.

## 1. Introduction

In recent years, many researchers have focused on catalysts employing supports composed of natural clay minerals, which are abundant, easily obtainable and inexpensive materials. Clay minerals are hydrous phyllosilicates [1]. The clay minerals belonging to the smectites group are used frequently. Each 2:1 layer of such clay mineral is of the (TOT) type consisting of two tetrahedral (T) Si-O sheets and an octahedral (O) Al-O/Al-OH sheet between them. The tetrahedral Si atoms are substituted by Al and/or a position of the octahedral atoms (Al or Mg) are substituted by atoms of lower oxidation number, generating the negative charges of the layers which are compensated by interlayer cations [2,3]. Most clay minerals contain iron in the amounts varying from traces to up to 30% mass [4]. Iron can substitute for Si in tetrahedral or for Al in octahedral sites. Structural iron in the octahedral site can exist in either Fe^2+^ or Fe^3+^ state. Experimental studies have indicated that electron transfer to/from clay mineral structure takes place through either edge sites, or through the basal surface [5]. Conversely, the computational atomistic models constructed using density functional theory supported electron transfer into octahedral Fe^3+^ sheets predominantly at the clay edges [6]. The structural iron content can be used to examine the extent of reduction and oxidation of Fe-bearing mineral clay [7]). The interlayer space, surface and edges of clay minerals provide sites to accommodate and stabilize Fe_2_O_3_ nanoparticles, thereby leading to the formation of a class of functional nanoparticle/clay minerals such as γ-Fe_2_O_3_/montmorillonite [8], α-Fe_2_O_3_/Na-montmorillonite [9]), Fe_2_O_3_/Fe_3_O_4_/vermiculite [10] and γ-Fe_2_O_3_/kaolinite [11]. The two-dimensional expandable layers of clay minerals provide useful hosts, which facilitate the introduction of catalytically active metal complexes [12]. Clay mineral, as supports of iron and iron oxide minerals, can also serve as heterogeneous catalysts in the Fenton-like reaction, which is propagated by the reduction of Fe^3+^ oxides to Fe^2+^ oxides under ultraviolet radiation [13].

Iron oxide is a transitional metal oxide crystallizing at different stoichiometric and crystalline structures, including wüstite (FeO), hematite (α-Fe_2_O_3_), maghemite (γ-Fe_2_O_3_) and magnetite (Fe_3_O_4_). Hematite is an n-type semiconductor (E_g_ = 2.1 eV) and has an advantage over the other conventional materials such as TiO_2_, ZnO, etc., for photocatalytic applications due to its narrow band gap of approximately 2.0–2.2 eV. Furthermore, hematite absorbs light up to 600 nm, collects up to 40% of the solar spectrum energy, is stable in most aqueous solutions (pH > 3), and is one of the cheapest semiconductor materials [14,15,16,17]. Hematite crystallizes in the hexagonal close-packed planes of oxygen anions arranged along the (001) plane with iron cations in the octahedral (001) basal plane. The arrangement of Fe^3+^ cations generates pairs of FeO_6_ octahedrons with two different Fe–O bond lengths [18]. The photocatalytic activity of the α- and γ-Fe_2_O_3_ nanoparticles of 25–55 nm in size was tested on the photocatalytic decomposition of H_2_S under visible light irradiation [19]. Similarly, α-Fe_2_O_3_ powder was tested on decomposition of aqueous solution of Rose Bengal dye [20] and Congo red dye [21]. The photocatalytic performance of α-Fe_2_O_3_ is limited by high recombination rate of electrons and holes, low diffusion lengths of holes (2–4 nm), and poor conductivity. The factors are influenced by the size and morphology of nanocrystals, their surface area, porosity, pH of the reaction medium, calcination temperature, reaction temperature, light intensity, and presence of oxidizing agents [17,19,22]. For example, the ammonia precipitant (NH_4_OH) provides spherical hematite particles whereas sodium hydroxide (NaOH) produces acicular hematite particles [23]. Calcination temperature of precipitates influences surface area of calcined material. α-Fe_2_O_3_ powders calcined at the 500 °C show a higher surface area and better photocatalytic activity than powders prepared at 600 °C [20]. The thermal evolution of the crystalline α-Fe_2_O_3_ by the chemical precipitation method is particularly attractive because of its low cost, high purity, short preparation time, high homogeneity, well-crystallized product and relatively low reaction temperature [24]. Most of Fe ions of photocatalysts were in fully oxidized states (Fe^3+^) detected by XPS analysis after annealing at temperature above 450 °C [25].

CO_2_ is a major component of the greenhouse gases contributing to global warming, and atmospheric pollution is the most serious of environmental problems. Nanocrystalline Fe_2_O_3_ can decompose CO_2_ at 400–600 °C to carbon nanotubes [15] and has been proven as a new visible photocatalyst towards the decontamination of NO_X_ gases [26] under UV-Vis light irradiation. An electron in the valence band acquires the energy of a photon to become a photogenerated electron (e^−^), which migrates to the conduction band and simultaneously leaves behind a photogenerated hole (h^+^), see Equation (1):

Fe_2_O_3_ + hν ⇒ e^−^ + h^+^ (1)

The pair of mobile charges initiate the redox process on the surface of Fe_2_O_3_ particle. The H_2_O molecule reacts with h^+^ to produce a hydroxyl radical, see Equation (2):

H_2_O + h^+^ ⇒ H^+^ + OH• (2)

Photocatalytic reduction of CO_2_ is one of the environmentally friendly methods that can be used to decrease CO_2_ emissions in the air using sunlight as the source of radiation. Photocatalytic hydrogenation of carbon dioxide is a promising technology that can convert carbon dioxide under ambient condition to sustainable fuels, such as methane or methanol. Among various proposed photocatalysts, TiO_2_ has been extensively studied over the past several decades for CO_2_ photocatalytic reduction because of its low cost and environmental friendliness [27,28,29,30,31].

This work presents a new type of photocatalyst on the clay mineral carrier. The structural, optical and photocatalytic properties of α-Fe_2_O_3_ nanoparticles/vermiculite clay materials, synthesized by the precipitation procedure from the FeCl_3_∙6H_2_O and NaOH precursors were studied after annealing samples at 500 °C and 700 °C for 4 h.

## 2. Materials and Methods

### 2.1. Materials and Sample Preparation

Raw vermiculite (layer charge −0.66, denoted as Ver) from Palabora mine, South Africa (supplied by Grena Co., Veselí nad Lužnicí, Czech Republic) was ball-milled in a cylindrical agate container (7 cm inner diameter) using 9 balls (10 mm diameter), 3 balls (20 mm diameter) and 2 balls (30 mm diameter) for 15 min at 550 rpm (agate planetary mill Fritsch Pulverisette 5). The size of the fraction ˂40 µm of the Ver was obtained by sieving and was used as a carrier of the nanoparticles. Pure α-Fe_2_O_3_ nanoparticles were synthesized with the chemical precipitation method from the FeCl_3_∙6H_2_O (iron chloride hexahydrate) precursor (supplied by Lach-Ner Co., Neratovice, Czech Republic). In this procedure, aqueous solution (c = 0.05 M) was prepared by dissolving 4 g of FeCl_3_∙6H_2_O in 100 mL of distilled water under magnetic stirring for 30 min at 70 °C. The precipitating agent (50 mL of NaOH aqueous solution, c = 2 M) was added gradually to maintain a pH value of 11 under magnetic stirring for 3 h at 70 °C. The resulting precipitates were collected and centrifuged at 6000 rpm and then washed several times with distilled water to free chlorides. The samples denoted as α-Fe_2_O_3__500 and α-Fe_2_O_3__700 were finally dried in air at 80 °C and calcined at 500 °C and 700 °C, respectively, for 4 h. Samples α-Fe_2_O_3__500/Ver and α-Fe_2_O_3__700/Ver were obtained using the identical procedure starting from the solution of FeCl_3_∙6H_2_O precursor (100 mL) and Ver (4 g) dispersed in precipitating agent NaOH aqueous solution (100 mL) under magnetic stirring for 3 h at 70 °C. Samples for analysis were then manually pulverized with an agate mortar and pestle.

### 2.2. Methods

The X-ray diffraction (XRD) analyses were carried out on the X-ray diffractometer Rigaku SmartLab (RIGAKU Corporation, Tokyo, Japan). The XRD patterns of powder samples on the Si-holder were recorded at the rotation speed of 15 rpm/min^−1^ in symmetrical Bragg–Brentano diffraction geometry in the 5–80° 2θ range with a step size of 0.01° and speed 0.5 deg/min^−1^. For the XRD measurement, CoKα radiation (λ1 = 0.178892 nm, λ2 = 0.179278 nm) at 40 kV and 40 mA, a detector D/teX Ultra 250 were used. The divergence of the primary X-ray beam was limited by 10 mm × 10 mm automatic divergence slits. Slits on the diffracted beam were set up to fixed values of 8 mm and 14 mm.

Scanning electron microscopy (SEM) Quanta FEG 450 (FEI Company, Hillsboro, OR, USA) with energy dispersive X-ray spectrometry (EDS) were used to characterize the morphology of hematite particles and the distribution of elements using EDS maps in samples. The samples were coated with a gold/palladium film to avoid an electrical charging. Backscatter electron (BSE) images were obtained using secondary and backscattered electron detectors.

Chemical elemental analysis was performed using the energy dispersive X-ray fluorescence (ED-XRF) spectrometer SPECTRO XEPOS (Spectro Analytical Instruments GmbH, Kleve, Germany) to determine amount of α-Fe_2_O_3_ anchored on vermiculite substrate. Each sample (4 g) in duplicate was mixed with the wax (0.9 g) and prepared in pellets using hydraulic pressing at 10 tons.

The specific surface area (S_BET_) was measured using the Sorptomatic 1990 instrument (Thermo Finnigan, Rodano, Italy). The Brunauer–Emmett–Teller (BET) N_2_ physisorption method was determined by employing the Advance Data Processing software according to the BET isotherm.

The diffuse reflectance UV–Vis spectra (DRS) were recorded using a UV-2600 spectrophotometer with an ISR-2600 integrating sphere attachment (Shimadzu Scientific Co., Tokyo, Japan) from 220 to 800 nm. The interval of sampling was set to 1 nm and the width of the slit to 2 nm at the integration sphere diameter of 60 nm, the external 2D detector and barium sulphate (BaSO_4_) powder pressed into the powder sample holder were used as reference material.

The PL spectra were recorded by a spectrometer FLSP920 Series (Edinburgh Instruments, Ltd., Livingston, UK) from 350 to 620 nm. The spectrometer was equipped with a 450 W Xenon lamp (Xe900) and a R928P type PMT detector. Spectrometer configuration was of the Czerny–Turner type: the excitation wavelength of 325 nm; the width 3.0 nm of the excitation slit, and 8.0 nm of the emission slit; dwell time was 0.5 s.

### 2.3. Molecular Simulation

Interlayer space arrangement of the Palabora vermiculite, as well as mutual α-Fe_2_O_3_/vermiculite interaction, was investigated by molecular modeling using a force field in the Biovia Materials Studio modeling environment (MS). The initial model of the Palabora vermiculite structure was prepared based on the crystal structure data a = 5.349 Å, b = 9.255 Å, c = 14.445 Å, α = 90°, β = 97.07°, γ = 90°, published by Shirozu and Bailey [32] and the crystallochemical formula (K^+^_0.46_Na^+^_0.03_Ca^2+^_0.09_) (Si_3.18_Al_0.79_Fe^3+^_0.03_) (Mg_2.7_Fe^3+^_0.2_Fe^2+^_0.08_) O_10_ (OH)_2_ (layer charge −0.66 e^−^) calculated from the chemical analysis of metal oxides concentrations, assuming that the trioctahedral structure of vermiculite is based on 22 negative layers resulting from O_10_(OH)_2_, cpfu. Periodic models of the vermiculite, having a corresponding crystallochemical formula (K^+^_11_Na^+^Ca^2+^_2_) (Si_76_Al_19_Fe^3+^) (Mg_66_Fe^3+^_4_Fe^2+^_2_) O_240_ (OH)_48_ (layer charge −16 e^−^), were prepared as a 6a × 4b × 1c supercell requiring rigid layers with fixed cell parameters a, b, γ [33]. These models were used to study the space arrangement of the interlayer material containing various amounts of interlayer cations and water molecules.

The strategy used to build models of vermiculite with α-Fe_2_O_3_ nanoparticles on its surface is based on the nonperiodic structure prepared from the 12a × 6b × 7c supercell of the Palabora vermiculite, having the crystallochemical formula of (K^+^_462_Na^+^_42_Ca^2+^_84_) (Si_3188_Fe^3+^_42_A_l798_) (Mg_2772_Fe^3+^_168_Fe^2+^_84_) O_10072_ (OH)_2016_ with a corresponding layer charge −672 e^−^ compensated by K^+^, Na^+^, and Ca^2+^ interlayer cations. The crystal structure of α-Fe_2_O_3_ was taken from the MS library (unit cell Fe_12_O_18_; a = b = 5.035 Å, c = 13.72 Å, α = β = 90°, γ = 120°). Two models of α-Fe_2_O_3_ nanoparticles (Fe_318_O_477_, their size about 3 nm) with crystallographic orientations (104) and (012) were built and then subsequently placed on the (001) surface and on the (100) surface (i.e., edge) of the vermiculite superstructure to study the nanoparticle–vermiculite interaction. The shape of nanoparticles in the models has no significant influence on the interaction with the clay mineral surface [34]. Although the atomistic models of the nanoparticles/phyllosilicate nanocomposites are always simplified real samples, they exhibit good agreement with experimental data, e.g., [33,34,35,36].

Atomic charges were calculated via charge equilibration method [37] in MS/Forcite module. The Universal force field (UFF) [38] was used to parameterize a wide variety of different atoms and atomic types present in the models. Smart algorithm (i.e., the cascade of Steepest descent, Conjugate gradient and Quasi-Newton algorithms as implemented in the MS/Forcite module) was used for the geometry optimization procedure with 50,000 iteration steps. Convergence criteria 1 × 10^−3^ kcal/mol and 5 × 10^−2^ kcal/mol/nm for energy and force, respectively, were used.

The basal distances in the optimized periodic models of the vermiculite interlayer space were obtained from the XRD patterns simulated in an MS/Reflex module. Conditions similar to the experimental measurements were used: Bragg–Brentano geometry, step size 0.05, CuKα source (λ = 1.540562 Å).

Interaction energy (E_int_; kcal/mol) for vermiculite/nanoparticle models were calculated according to Formula (3):

E_int_ = E_tot_ − (E_tot,NP_ + E_tot,VER_), (3)

where E_tot_ is a total potential energy of the optimized vermiculite/nanoparticle model, E_tot,NP_ is a total potential energy of the nanoparticle in the optimized model, and E_tot,VER_ is a total potential energy of the vermiculite in the optimized model.

### 2.4. Photocatalytic Test

Photocatalytic tests were performed in a batch mixed photoreactor (stainless steel, volume 348 mL). Reaction mixture contained 100 mL of NaOH (0.2 M) with a photocatalyst (0.1 g) and was saturated by helium or CO_2_, to purge the air and to saturate the solution. The reaction suspension was investigated for each photocatalyst behavior for water splitting and thereafter for the CO_2_ photocatalytic reduction, respectively. An 8W Hg lamp (Ultra-Violet Products Inc., USA, 11SC-1, λmax = 254 nm, average light intensity = 4.5 mW/cm^2^, see Figure S1) was used as the irradiation source and was placed on a quartz glass window in the top of the photoreactor. The reactor was tightly closed (the pressure was maintained at 110 kPa) and before the start of the reaction (switching on the UV lamp), a gaseous sample was taken (at time 0 h) through septum by syringe. The all gaseous samples were analyzed by a gas chromatograph equipped with a barrier discharge ionization detector (Tracera GC—2010 plus, Shimadzu). The reaction mixture was irradiated at certain time intervals (0–14 h) and samples were taken at 8, 10, 12 and 14 h for analysis. All of the measurements were reproducibly measured.

Blank reactions tests (in the dark with the photocatalyst and UV-illuminated without the photocatalyst) were performed to ensure that the hydrogen and carbon monoxide production was due to the presence of a photocatalyst.

## 3. Results and Discussion

The development of Fe_2_O_3_ nanoparticles can be explained by the chemical reactions concerned and the crystal growth behaviors of iron oxide. The synthesis of Fe_2_O_3_ NPs was performed using ferric chloride (FeCl_3_) and NaOH under continuous stirring at 70 °C. NaOH maintains the pH value as well as bringing hydroxyl ions to the solution. The FeCl_3_ reacts with NaOH and forms FeO(OH) according to the chemical reactions (4)–(6).

NaOH_(aq)_ → Na^+^_(aq)_ + OH^−^_(aq)_ (4)

FeCl_3(s)_ → Fe^3+^_(aq)_ + 3Cl^−^_(aq)_ (5)

Fe^3+^_(aq)_ + 2OH^−^_(aq)_ → FeO(OH)_(aq)_ + H^+^ (6)

The FeO(OH) dissociates to the formation of Fe_2_O_3_ nuclei according to the reactions (7)–(8):

FeCl_3(aq)_ + 2NaOH_(aq)_ → FeO(OH)_(aq)_ + 2Na^+^_(aq)_ + H^+^_(aq)_ + 3Cl^−^_(aq)_ (7)

2FeO(OH)_(aq)_ → Fe_2_O_3(s)_ + H_2_O (8)

The reaction under the appropriate heating conditions increases the concentration of Fe_2_O_3_ nuclei, which are the basis of the construction of the desired nanoparticles.

### 3.1. X-ray Diffraction Analysis (XRD)

**Figure 1:**
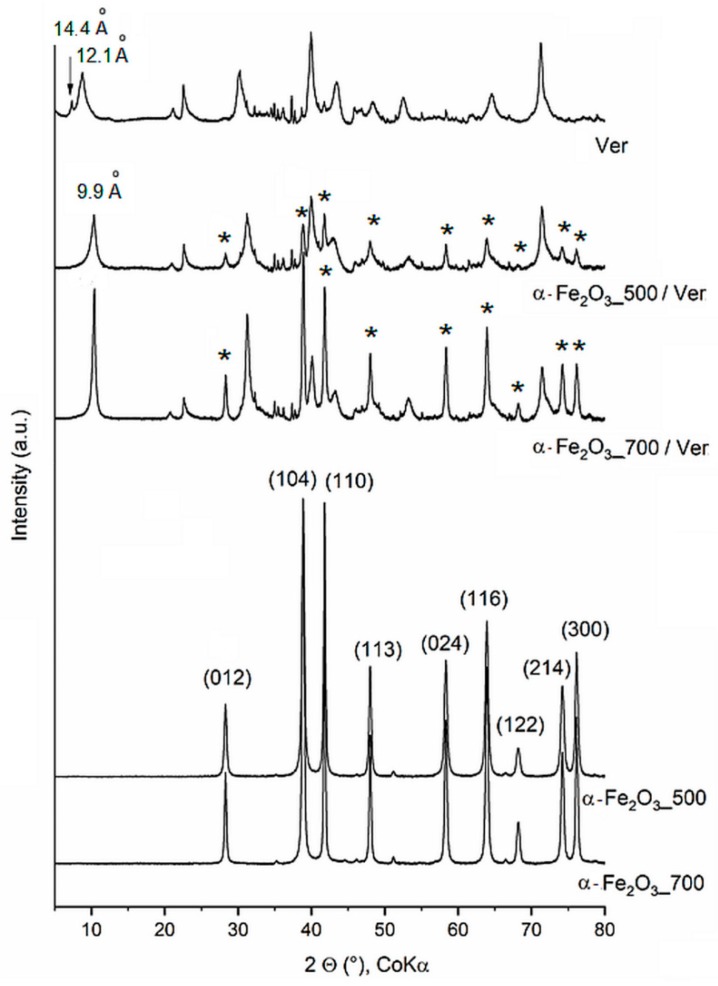
XRD patterns of α-Fe_2_O_3_ and α-Fe_2_O_3_/Ver samples calcined at 500 °C and 700 °C (peaks of α-Fe_2_O_3_ are marked by asterisk).

The irrational basal peak series in the XRD pattern of raw vermiculite (Ver) (Figure 1) suggest mixed layering of vermiculite-mica-like layers, having interlayer cations at different water hydration states. The d-values of 14.4 Å and 12.1 Å are consistent with the mixture of vermiculite water bilayers and interstratified dehydrated phase, respectively [39]. The change in intensity and the position of basal reflections at d = 9.9 Å of vermiculite in α-Fe_2_O_3__500/Ver and α-Fe_2_O_3__700/Ver agree with the interlayer space containing non-hydrated cations. All peaks of the iron oxide precipitated and annealing at 500 °C and 700 °C were assigned according to PDF card No. 01-076-4579 to pure hematite, α-Fe_2_O_3_ (Figure 1).

The peaks of α-Fe_2_O_3__700 are more intense and narrower in comparison with the peaks of α-Fe_2_O_3__500, indicating higher crystallinity due to the formation of larger nanoparticles and due to agglomeration of the smaller ones. The mean size of α-Fe_2_O_3_ crystallites (Table 1) was calculated from the broadening of the diffraction lines (012) and (104). The Scherrer equation [40] explains the physical broadening of XRD lines in terms of a limited size of coherently diffracting domains (crystallites), which leads to a line broadening that is identical for all reciprocal lattice points throughout the reciprocal space. This means that the mean crystallite size D can be calculated from the physical part of the reciprocal line width expressed in the units of the reciprocal space, β, as D = K/β, or as D = K λ/(B cos θ) if the peak breadth (B) is expressed in the angular scale (in radians) [41]. In these equations, K is a constant, depending on the shape of the crystallites, λ is the wavelength of the X-rays and θ is a half of the diffraction angle. For the spherical shape of the crystallites, the factor K is equal to 0.9 [42]. The mean crystallite sizes (D in Table 1) of nanoparticles α-Fe_2_O_3_ were calculated as approximately D = 24 nm and 31 nm for α-Fe_2_O_3__500 and α-Fe_2_O_3__700, respectively, which are comparable with reported D = 19 nm for α-Fe_2_O_3_ at 500 °C and D = 27 nm for α-Fe_2_O_3_ at 650 °C [20].

**Figure 2:**
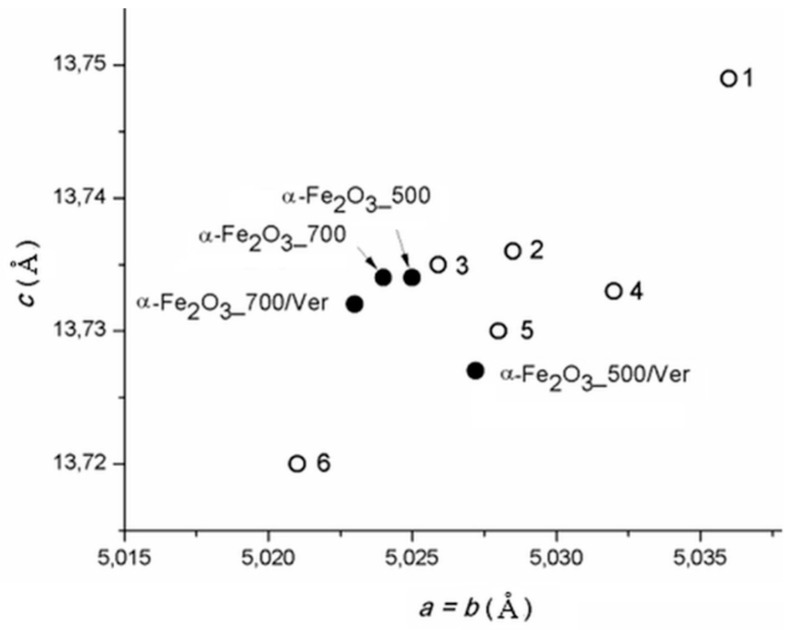
Lattice parameters *a* vs. *c* of α-Fe_2_O_3_ calculated for studied samples and in PDF cards: No. 00-033-0664 (1), No. 01-079-0007 (2), No. 01-076-4579 (3), No.01-089-0599 (4), No. 00-001-1053 (5) and No. 01-089-8103 (6).

The calculated lattice parameters *a* vs. *c* of pure α-Fe_2_O_3_ nanoparticles and bounded in/on α-Fe_2_O_3_/vermiculite clay materials ((Figure 2, Table 1) are supplemented by the parameters of α-Fe_2_O_3_ in the PDF cards: No. 00-033-0664 (marked as 1), No. 01-079-0007 (marked as 2), No. 01-076-4579 (marked as 3), No.01-089-0599 (marked as 4), No. 00-001-1053 (marked as 5) and No. 01-089-8103 (marked as 6). Although the calculated cell dimensions vary in the range of their deviations by approximately 0.01 Å, the parameters of pure α-Fe_2_O_3_ and α-Fe_2_O_3_ anchored on the vermiculite are not the same, and the influence of vermiculite carrier on the symmetry of the cell parameters during crystallization of hematite can be assumed.

### 3.2. Scanning Electron Microscopy (SEM)

**Figure 3:**
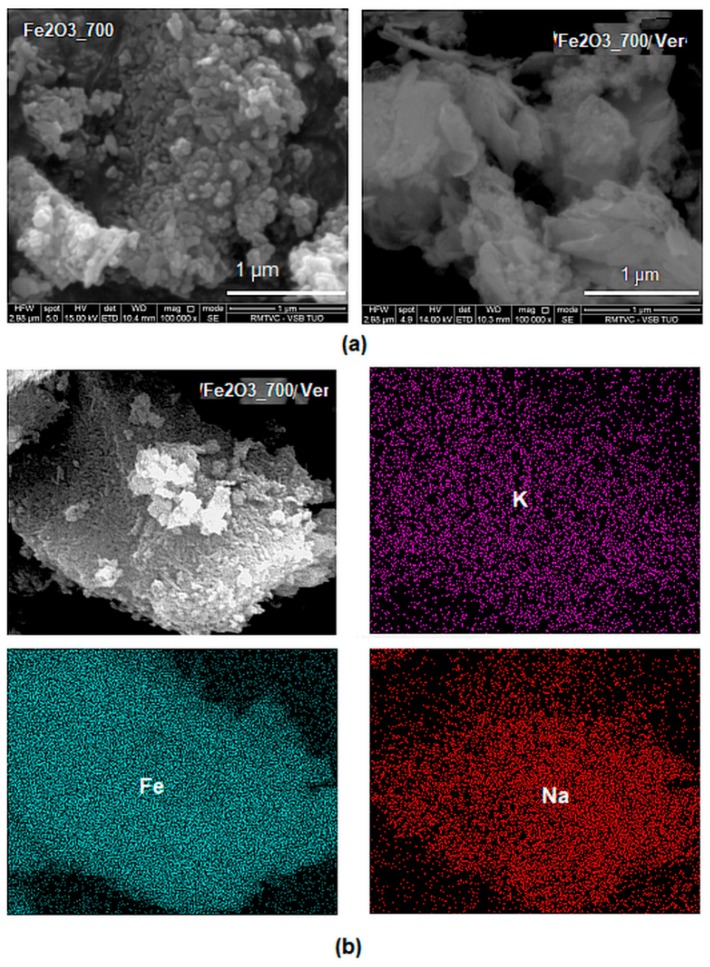
Backscattered electron (BSE) images: (**a**) α-Fe_2_O_3_ nanoparticles after precipitation in water solution and an aqueous dispersion of vermiculite (sample α-Fe_2_O_3__700/Ver) calcined at 700 °C; (**b**) EDS maps of elements Fe, K and Na on the surface of vermiculite flake α-Fe_2_O_3__700/Ver.

The ED-XRF analysis of sample α-Fe_2_O_3_/Ver determined a Fe_2_O_3_ 27.5% mass. The BSE images of α-Fe_2_O_3_ nanoparticles (Figure 3) showed acicular morphology provided by the NaOH precipitant after calcination (Figure 3a), as already described in the literature, e.g., [23]. The distributions of nanocrystals were irregular, and their size ranged from sole nanoparticles up to nanoparticles clusters. EDS maps of elements Fe and Na (originating from the precipitation precursors) and K bound in the vermiculite structure (Figure 3b) revealed their homogeneous distribution on the surface of vermiculite flake.

### 3.3. Molecular Simulations

**Figure 4:**
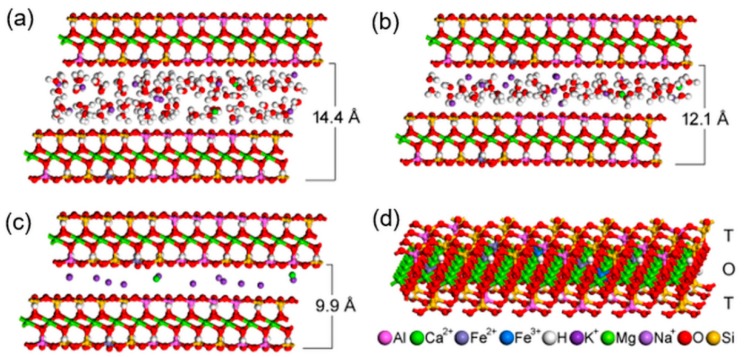
Four models of vermiculite interlayer space arrangement: (**a**) corresponding to the basal distance c = 14.4 Å; (**b**) corresponding to the basal distance c = 12.1 Å; (**c**) corresponding to the basal distance c = 9.9 Å in vermiculite thermally treated at 500 °C and 700 °C; (**d**) Model of a single 2:1 layer showing the positions of atoms in the tetrahedral (T) and octahedral (O) sheets. The color legend for all atoms occurring in the models is below the image.

The space arrangement of the interlayer content in the optimized vermiculite models, the basal distances of which are calculated from simulated diffractograms, are in best agreement with the experimental data (Figure 4a–c). The two basal reflections of the original natural vermiculite with the interlayer values of 14.4 Å and 12.1 Å (Figure 1) correspond to the two water layers and one water layer around interlayer cations, respectively (compare Figure 4a,b). The stability of the optimized structures is almost the same, the total potential energy is −71,009 kcal/mol (Figure 4a) and −70,900 kcal/mol (Figure 4b). The decrease in basal distance to 9.9 Å after thermal treatment (Figure 1) is the result of the loss of interlayer water molecules (Figure 4c). Only a negligible decrease in stability of this anhydrous model to −70,355 kcal/mol (Figure 4c) can be explained by the intrinsic property of the interlayer cations to attract water molecules and to form hydration shells.

**Figure 5:**
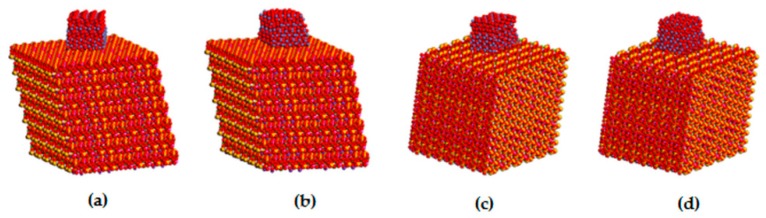
Optimized structure models of: (**a**) α-Fe_2_O_3_(012)/VER(001), (**b**) α-Fe_2_O_3_(104)/VER(001), (**c**) α-Fe_2_O_3_(012)/VER(100), and (**d**) α-Fe_2_O_3_(104)/VER(100).

The structure of a single vermiculite layer with tetrahedral and octahedral substitutions, which are the source of a negative layer charge, is demonstrated in Figure 4d. Molecular modeling of α-Fe_2_O_3_ nanoparticles adjacent via selected crystallographic planes (012) and (104) to the surface and the edge of vermiculite showed the possibility of anchoring the nanoparticles to the Palabora vermiculite (Table 2). Stability of all four models is very similar, with the total potential energy on average being −210,987 kcal/mol, while the interaction energy shows that (012) orientation of α-Fe_2_O_3_ is preferred, both on the surface and the edge of the vermiculite (Table 2). Higher interaction energies for models containing α-Fe_2_O_3_ nanoparticles at the vermiculite edge are caused by a higher roughness of the edge compared to the surface. This result does not necessarily mean a lower occurrence of α-Fe_2_O_3_ nanoparticles at the vermiculite edges in the real sample, because nanoparticles on the edges tend to be smaller than on the surface, so the contact area between nanoparticle and edge increases. This problem was discussed in our earlier study focused on a similar nanocomposite system of metal oxide/phyllosilicate-type TiO_2_/kaolinite [43]. The optimized structures of all four models, the energies of which are listed in Table 2, are displayed in Figure 5.

### 3.4. UV-Vis DRS

**Figure 6:**
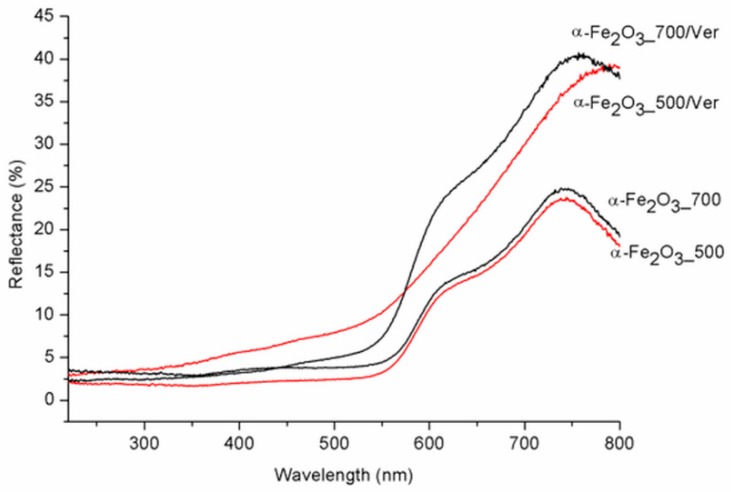
Diffuse reflectance spectra of pure α-Fe_2_O_3_ nanoparticles and α-Fe_2_O_3_ nanoparticles/Ver samples for different temperatures.

UV-Vis diffuse reflectance spectroscopy is useful for identifying diverse types of iron oxides and to obtain information about the optical properties. As a rule, colored Fe oxides show a strong absorption in the ultraviolet and blue spectral regions, except for strong reflection in the red and infrared region [44]. The DRS spectra of α-Fe_2_O_3__500 and α-Fe_2_O_3__700 are in good agreement with the spectrum of room-temperature hematite powder characterized by Yamanoi et al. [45]. The spectra in Figure 6 show a nearly constant reflectivity in the range 400–550 nm, a shoulder near 620 nm, and a reflectivity maximum near 750 nm. The region between 700 nm and 800 nm with low absorption shows very high reflectance. These bands shift to longer wavelengths (red shift) as the temperature increases with increasing particle size. While the DRS spectra of α-Fe_2_O_3__700/Ver and α-Fe_2_O_3__700 are similar, DRS spectrum of α-Fe_2_O_3__500/Ver is different and corresponds with the deflection of unit cell parameters calculated for α-Fe_2_O_3_ in α-Fe_2_O_3__500/Ver sample (Table 1, Figure 2). The calculated absorption intensities (Kubelka–Munk functions [46]) suggest that this temperature dependence of the hematite reflectance spectra can mainly be explained by a change in band gap absorption edges for a semiconductor. To determine the values of the direct/indirect bands gap energy (*E_g_*), reflectance was recalculated into the dependence of Kubelka–Munk function (F(R_∞_)) on the absorption energy using the Equation F(R_∞_) = (1  −  R_∞_)^2^/(2·R_∞_), where R_∞_ is the diffuse reflectance (R_∞_ = R_sample_/R_standard_) from a semi-infinite layer. The band gap energies were calculated using the Tauc’s relation *αhν* = A (*hν* − *E_g_*)*^n^*, where *α* is adsorption coefficient, *hν* is the incident photon energy, A is constant and n = 2 for direct band gap energy for these materials [47].

**Figure 7:**
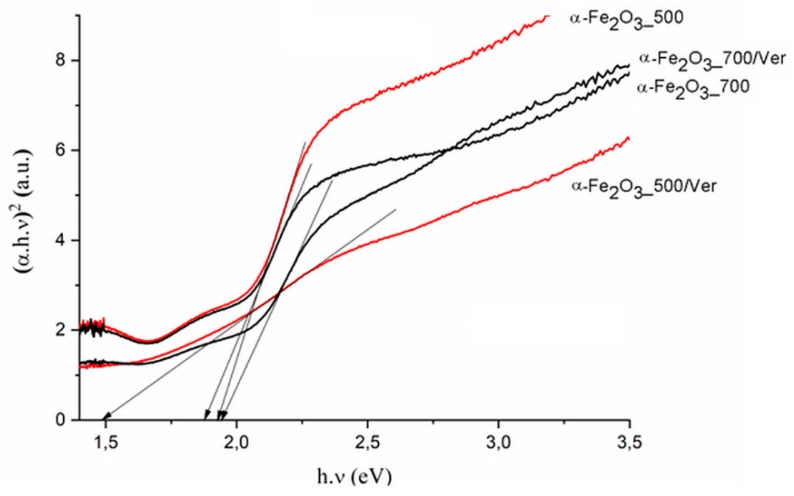
Tauc plot of (*αhν*)^2^ versus photon energy (*hν*) for pure α-Fe_2_O_3_ nanoparticles and α-Fe_2_O_3_ nanoparticles/Ver samples at 500 °C and 700 °C.

The direct band gap energy (B_g_ values) for pure α-Fe_2_O_3_ were reported in the range from 1.88 to 1.93 eV, depending on its crystalline state and methods of preparation (e.g., [48]). The B_g_ values for α-Fe_2_O_3_ nanoparticles (Table 3, Figure 7) and the reduced gap energy B_g_ of about 0.05 eV at calcination temperature 700 °C in comparison with 500 °C are caused by crystallites larger than approximately 7 nm at α-Fe_2_O_3__700 (Table 1). These characteristics are similar to the α-Fe_2_O_3_ crystallite size of 33 nm and 21 nm influencing the B_g_ values of 1.94 and 2.1 eV, respectively, as reported by Maji et al. [20].

In spite that the crystallite size of α-Fe_2_O_3_ nanoparticles on vermiculite not changing fundamentally in comparison to their parent free samples (Table 1), the maximum difference of the band gap energy value B_g_ of about 0.45 eV was determined between α-Fe_2_O_3__700/Ver (B_g_ = 1.94 eV) and α-Fe_2_O_3__500/Ver (B_g_ = 1.49 eV) (Table 3).

The variation of the band gap energies observed in the hematite were explained by the defect states, cation/anion vacancies, and interstitials with energy located within the band gap energy (e.g., Mathevula et al. [47]). In our samples, B_g_ values were affected by vermiculite more intensively than by the calcination temperatures 500 °C and 700 °C. During calcination, heating from about 500 °C to 850 °C, when hematite nanoparticles were crystallized, vermiculite exfoliation was associated with a sudden release of water molecules between the silicate layers and the hydroxyl water gradually releasing [49]. At 500 °C and 700 °C, interlayer water molecules no longer existed, and vermiculite structure changed to a 1 Å mica-like structure (Figure 1 and Figure 4c). Crystallization of large particles at higher temperature was accompanied by the S_BET_ value (Table 3), and reduced about 68% in α-Fe_2_O_3__700 in comparison with α-Fe_2_O_3__500 and about 50% in α-Fe_2_O_3__700/Ver in comparison with α-Fe_2_O_3__500/Ver.

### 3.5. Photoluminiscence (PL)

**Figure 8:**
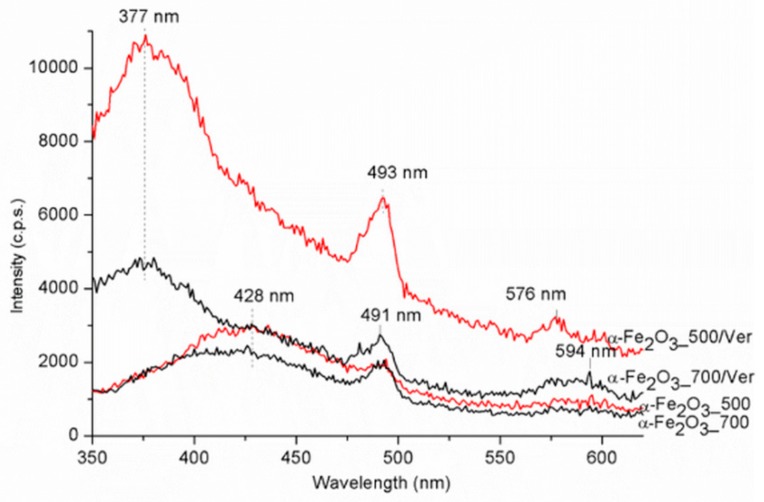
Photoluminescence spectra for α-Fe_2_O_3_ nanoparticles pure and attached on vermiculite annealed at 500 °C and 700 °C.

The PL spectra were carried out in the range of 350–700 nm (Figure 8). The maximum emission band for samples α-Fe_2_O_3__700 and α-Fe_2_O_3__500 centered around 428 nm is red shifted to about 377 nm for samples α-Fe_2_O_3__500/Ver and α-Fe_2_O_3__700/Ver. The strong photoluminescence was attributed to the electron transition, from the donor level formed by oxygen vacancies to the acceptor level [50,51]. The band centered around 493 nm was ascribed to the charge transfer transition between the iron ions [52]. A weak band at about 594 nm for samples α-Fe_2_O_3__700 and α-Fe_2_O_3__500 is red shifted to about 576 nm for samples α-Fe_2_O_3__500/Ver and α-Fe_2_O_3__700/Ver. This emission band can be attributed to the Fe^3+^ ligand field transition [47].

### 3.6. Photocatalytic Test

**Figure 9:**
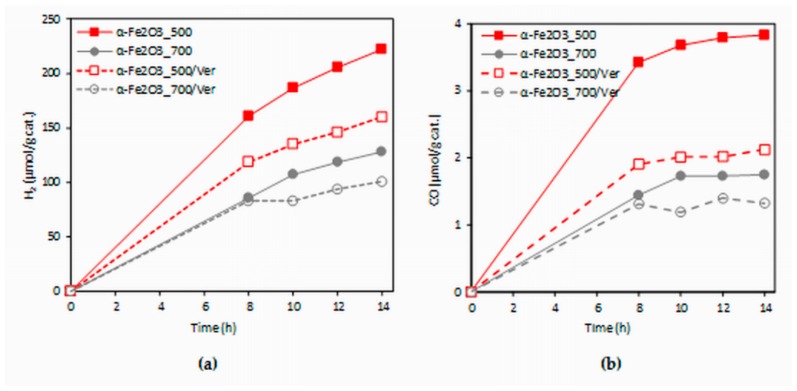
Time dependence of yields of: (**a**) H_2_ and (**b**) CO during the CO_2_ photocatalytic reduction in the presence of investigated photocatalysts.

The dependence of hydrogen and carbon monoxide yields of the CO_2_ photocatalytic reduction on the irradiation time (0–14 h) in the present investigation is shown in Figure 9. The formation of hydrogen ranged in the order of 100 and carbon monoxide in the order of units of µmol/g. The yields of products increased with irradiation time. The photocatalysts were measured repeatedly and all photocatalysts proved the photocatalytic stability (changes in photocatalytic activity did not occur in repeated use, see Figure S2).

**Figure 10:**
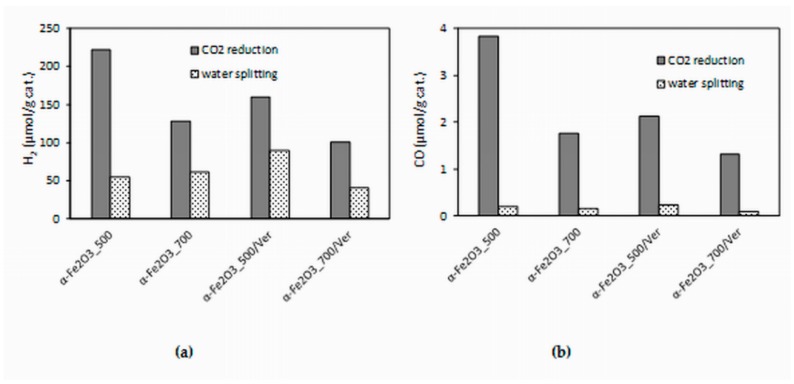
Dependence of yields of: (**a**) H_2_ and (**b**) CO (after 14 h of irradiation) during CO_2_ photocatalytic reduction and water splitting in the presence of investigated photocatalysts.

The experimental data from the longest irradiation time (14 h) were chosen for comparison, because all of the formed products yields were the most accurate. The effect of different photocatalysts on the yields of all products (hydrogen and carbon monoxide) is depicted in Figure 10. Both hydrogen yields and carbon monoxide yields in the presence of the α-Fe_2_O_3__500 photocatalyst were almost twice as high as that of the α-Fe_2_O_3__700 photocatalyst. Decrease of yields of products H_2_ and CO in the presence of α-Fe_2_O_3__500/Ver was 28% and 44%, respectively. However, it is necessary to note that α-Fe_2_O_3__500/Ver contained only 27.5% mass of α-Fe_2_O_3_. In view of this fact, the obtained results are promising.

The higher photocatalytic activity of α-Fe_2_O_3__500 photocatalyst in comparison with α-Fe_2_O_3__700 photocatalyst can be caused by a slightly higher specific surface area (25 m^2^/g and 17 m^2^/g, respectively) and smaller crystallite size (24 nm and 31 nm, respectively). Also, the amount of oxygen vacancies, which was higher in α-Fe_2_O_3__500 photocatalyst, played a significant role (Figure 8).

The photocatalysis is based on the formation of generated electrons and holes under light irradiation. The generated electron can migrate from the valence band (VB) of the semiconductor to the conduction band (CB), leaving behind a hole in the VB. If these charges are separated, they can migrate to the surface of the semiconductor and participate in redox reaction with adsorbed species. For this reason, it is important to perceive the role of oxygen vacancies as electron acceptors during the photocatalytic reaction and temporary traps of electrons to decrease the recombination of electrons and holes [53].

## 4. Conclusions

In this study, α-Fe_2_O_3_ nanoparticles/vermiculite clay photocatalysts were prepared and characterized. The molecular simulation showed the space arrangement of the interlayer content in the optimized vermiculite models to be in good agreement with the experimental data. The photocatalytic activity of prepared photocatalysts decreased in the order α-Fe_2_O_3__500 ˃ α-Fe_2_O_3__500/Ver ˃ α-Fe_2_O_3__700 ˃ α-Fe_2_O_3__700/Ver. Higher photocatalytic activity was associated with smaller crystallite size, higher specific surface area and a higher amount of oxygen vacancies, promoting better separation of electrons and holes. Based on the correlation between physicochemical properties and the photocatalytic activity of the investigated photocatalysts, it can be concluded that calcination at 500 °C in comparison with calcining at 700 °C provides structural and optical properties of α-Fe_2_O_3__500 and α-Fe_2_O_3__500/Ver more suitable for photocatalytic applications.

## Figures and Tables

**Table 1:** α-Fe_2_O_3_ nanoparticles: the mean coherent crystallite size and lattice parameters.

Sample	Crystallite Size D (nm)	Lattice *a* (Å)	Parameters *c* (Å)
α-Fe_2_O_3__500	24 ± 2	5.025 ± 0.008	13.734 ± 0.040
α-Fe_2_O_3__700	31 ± 3	5.024 ± 0.008	13.732 ± 0.038
α-Fe_2_O_3__500/Ver	23 ± 1	5.027 ± 0.009	13.727 ± 0.046
α-Fe_2_O_3__700/Ver	30 ± 1	5.023 ± 0.008	13.733 ± 0.043

**Table 2:** Total potential energies (E_tot_) and binding energies (E_b_) for optimized α-Fe_2_O_3_/vermiculite models. Energies are expressed in kcal/mol.

Model	E_tot_	E_b_
α-Fe_2_O_3_(012)/VER(001)	−212,431	−10,996
α-Fe_2_O_3_(104)/VER(001)	−210,605	−8202
α-Fe_2_O_3_(012)/VER(100)	−209,979	−7779
α-Fe_2_O_3_(104)/VER(100)	−210,931	−6928

**Table 3:** Band gap energies (B_g_) and specific surface area (S_BET_) of the studied samples.

Sample	B_g_ (eV)	S_BET_ (m^2^g^−1^)
α-Fe_2_O_3__500	1.93	25
α-Fe_2_O_3__700	1.88	17
α-Fe_2_O_3__500/Ver	1.49	59
α-Fe_2_O_3__700/Ver	1.94	30

## Supplementary Materials

The following are available online at https://www.mdpi.com/1996-1944/12/11/1880/s1, Figure S1: Spectrum of the Hg lamp; Figure S2: Production of hydrogen during the photocatalytic reduction of CO_2_ in presence α-Fe_2_O_3__500 in the three cycles.
