# Supplementary material for: α-Fe2O3 Nanoparticles/Vermiculite Clay Material: Structural, Optical and Photocatalytic Properties
Source: Materials (Basel). 2019 Jun 11;12(11):1880. doi: 10.3390/ma12111880 (PMC6600952; doi:10.3390/ma12111880)
Supplement: Supplementary file 1 [file materials-12-01880-s001.pdf]

materials

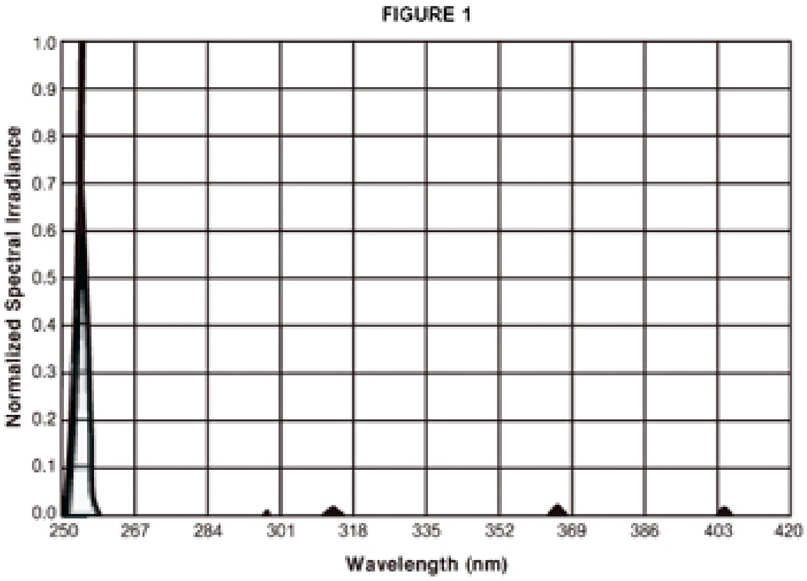

Figure S1. Spectrum of the Hg lamp.

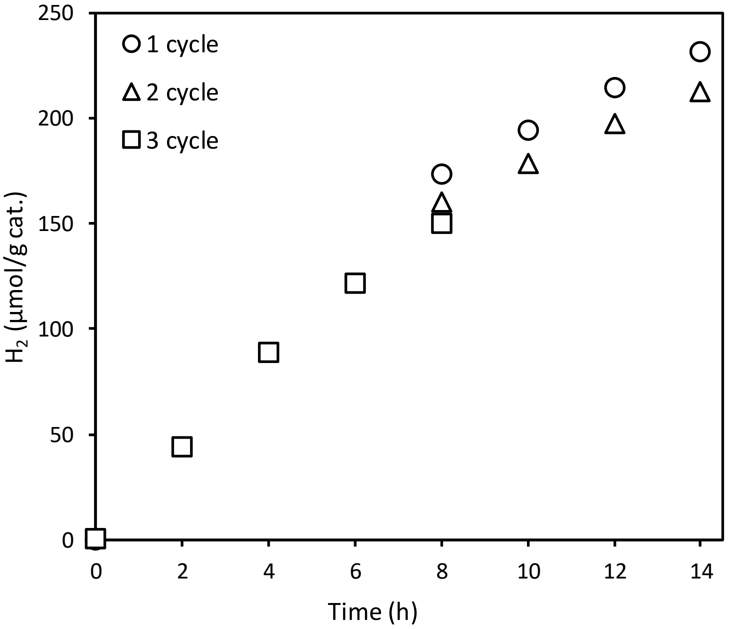

Figure S2. Production of hydrogen during the photocatalytic reduction of CO_2 in presence α-Fe_2O_3_500 in the three cycles.
